# The Interplay between Chondrocyte Redifferentiation Pellet Size and Oxygen Concentration

**DOI:** 10.1371/journal.pone.0058865

**Published:** 2013-03-15

**Authors:** Betul Kul Babur, Parisa Ghanavi, Peter Levett, William B. Lott, Travis Klein, Justin J. Cooper-White, Ross Crawford, Michael R. Doran

**Affiliations:** 1 Stem Cell Therapies Laboratory, Institute of Health and Biomedical Innovation, Faculty of Health, Queensland University of Technology and Translational Research Institute, Brisbane, Australia; 2 Medical Device Domain, Institute of Health and Biomedical Innovation, Queensland University of Technology, Brisbane, Australia; 3 Tissue Engineering and Microfluidics Laboratory, Australian Institute for Bioengineering and Nanotechnology, The University of Queensland, St. Lucia, Brisbane, Australia; 4 Mater Medical Research Institute, Brisbane, Australia; The Ohio State University, United States of America

## Abstract

Chondrocytes dedifferentiate during *ex vivo* expansion on 2-dimensional surfaces. Aggregation of the expanded cells into 3-dimensional pellets, in the presence of induction factors, facilitates their redifferentiation and restoration of the chondrogenic phenotype. Typically 1×10^5^–5×10^5^ chondrocytes are aggregated, resulting in “macro” pellets having diameters ranging from 1–2 mm. These macropellets are commonly used to study redifferentiation, and recently macropellets of autologous chondrocytes have been implanted directly into articular cartilage defects to facilitate their repair. However, diffusion of metabolites over the 1–2 mm pellet length-scales is inefficient, resulting in radial tissue heterogeneity. Herein we demonstrate that the aggregation of 2×10^5^ human chondrocytes into micropellets of 166 cells each, rather than into larger single macropellets, enhances chondrogenic redifferentiation. In this study, we describe the development of a cost effective fabrication strategy to manufacture a microwell surface for the large-scale production of micropellets. The thousands of micropellets were manufactured using the microwell platform, which is an array of 360×360 µm microwells cast into polydimethylsiloxane (PDMS), that has been surface modified with an electrostatic multilayer of hyaluronic acid and chitosan to enhance micropellet formation. Such surface modification was essential to prevent chondrocyte spreading on the PDMS. Sulfated glycosaminoglycan (sGAG) production and collagen II gene expression in chondrocyte micropellets increased significantly relative to macropellet controls, and redifferentiation was enhanced in both macro and micropellets with the provision of a hypoxic atmosphere (2% O_2_). Once micropellet formation had been optimized, we demonstrated that micropellets could be assembled into larger cartilage tissues. Our results indicate that micropellet amalgamation efficiency is inversely related to the time cultured as discreet microtissues. In summary, we describe a micropellet production platform that represents an efficient tool for studying chondrocyte redifferentiation and demonstrate that the micropellets could be assembled into larger tissues, potentially useful in cartilage defect repair.

## Introduction

Cartilage is an avascular tissue with poor regenerative capacity. Existing surgical repair strategies are limited in their efficacy [1], [2], [3], and largely function only to delay the onset of osteoarthritis [4], [5]. It is envisaged that autologous cell-based therapies will overcome these regenerative barriers, enabling defect repair and restoration of long-term joint function [6], [7]. However, in practice, cell-based therapies have demonstrated only modest efficacy relative to less complex and less costly treatment protocols such as microfracture [8], [9]. Nevertheless, the capacity of cell-based therapies to deliver more cells of an appropriate phenotype into defect sites is seen as a unique feature that will ultimately enable their efficacy. Notably, manufacturing this ideal cell population remains a challenge [10].

Clinically approved cell-based cartilage defect strategies utilize autologous chondrocytes harvested from non-weight bearing regions of the joint targeted for repair [6], [11]. Selection of articular chondrocytes as a starting population is rational, as these cells have a phenotype appropriate for articular cartilage tissue formation. However, this “optimal” phenotype is lost when the finite number of donor chondrocytes is expanded using traditional 2-dimensional (2D) tissue culture methodologies [12], [13]. A number of research groups have explored alternatives to conventional 2D expansion processes [14], [15], [16], but avoiding chondrocyte dedifferentiation whilst also achieving the necessary expansion in a clinically relevant time frame has not yet been achieved.

During *in vitro* studies, expanded chondrocytes are commonly redifferentiated through the aggregation of 1×10^5^–5×10^5^ cells into a 3-dimensional (3D) pellet in the presence of TGF-ß or other induction factors [17], [18], [19], [20], [21], [22]. The resulting “macropellet” is macroscopic, having diameters of 1–2 mm. Significant diffusion gradients develop over such length-scales, and as a result the redifferentiation phenotype and matrix deposition vary radially through the pellet [18], [23], [24], [25]. Despite this artifact, macropellet cultures remain the gold standard for studying chondrocyte redifferentiation *in vitro,* and in recent clinical trials they have been directly implanted into articular cartilage defects to facilitate tissue regeneration by co.don® AG (Teltow, Germany) [25], [26]. Macropellets’ popularity as a redifferentiation platform, and now potentially as a clinical tool, reflects the simple and robust methods used in their manufacture. The cells are easily pelleted via centrifugation in polypropylene tubes or v-bottom plates. Whilst being an inexpensive process, the heterogeneous product derived from pellet cultures limits our capacity to investigate and optimize redifferentiation mechanisms for clinical application.

In previous work we outlined how a commercial microwell product could be utilized to manufacture thousands of micropellets (166 cells each, diameters of ∼100 µm each) of mesenchymal stem/stromal cells (MSC) and subsequently differentiate them into chondrocytes [27]. The reduced diameter of the micropellets mitigated diffusion gradients, enhanced MSC chondrogenic differentiation and generated a more uniform cell product [27]. We reasoned that a similar strategy should also enhance chondrocyte redifferentiation, and that optimized chondrocyte micropellets should be capable of subsequent assembly into larger tissues, thereby demonstrating their potential in tissue engineering applications. To further optimize the redifferentiation process, and to better understand the role of hypoxia in relation to pellet dimension, redifferentiation studies were performed in hypoxic (2% O_2_) and normoxic (20% O_2_) atmospheres.

Identifying a cost effective platform for micropellet formation is essential for routine and thorough micropellet experimentation. To address this need, an in-house process for the manufacture of a microwell platform from polydimethylsiloxane (PDMS) was developed. This custom PDMS microwell platform was then surface modified with an electrostatic multilayer of hyaluronic acid (HA) and chitosan (CHI) to promote micropellet formation. Such surface modification was essential to prevent chondrocyte spreading on the PDMS, and the HA/CHI multilayer ensured robust micropellet formation. Using this platform, the redifferentiation of 2×10^5^ 2D-expanded chondrocytes, either assembled into single macropellets or into 1200 micropellets (166 cells each), in 2% and 20% oxygen atmospheres were contrasted over 14 days. Macropellets and micropellets were characterized for metabolic activity, total sulfated glycosaminoglycan (sGAG), DNA production, gene expression and histology. In subsequent experiments, the optimal micropellet manufacturing protocol (2% O_2_) was utilized to characterize the capacity of micropellets of different maturity to amalgamate into a single cartilage tissue. Micropellets from day 4, 8, 11 or 14 cultures were amalgamated up to 21 days, and integration was assessed via histology.

## Materials and Methods

All materials were purchased from SIGMA-ALDRICH® unless otherwise stated.

### Fabrication of Microwell Surface

**Figure 1:**
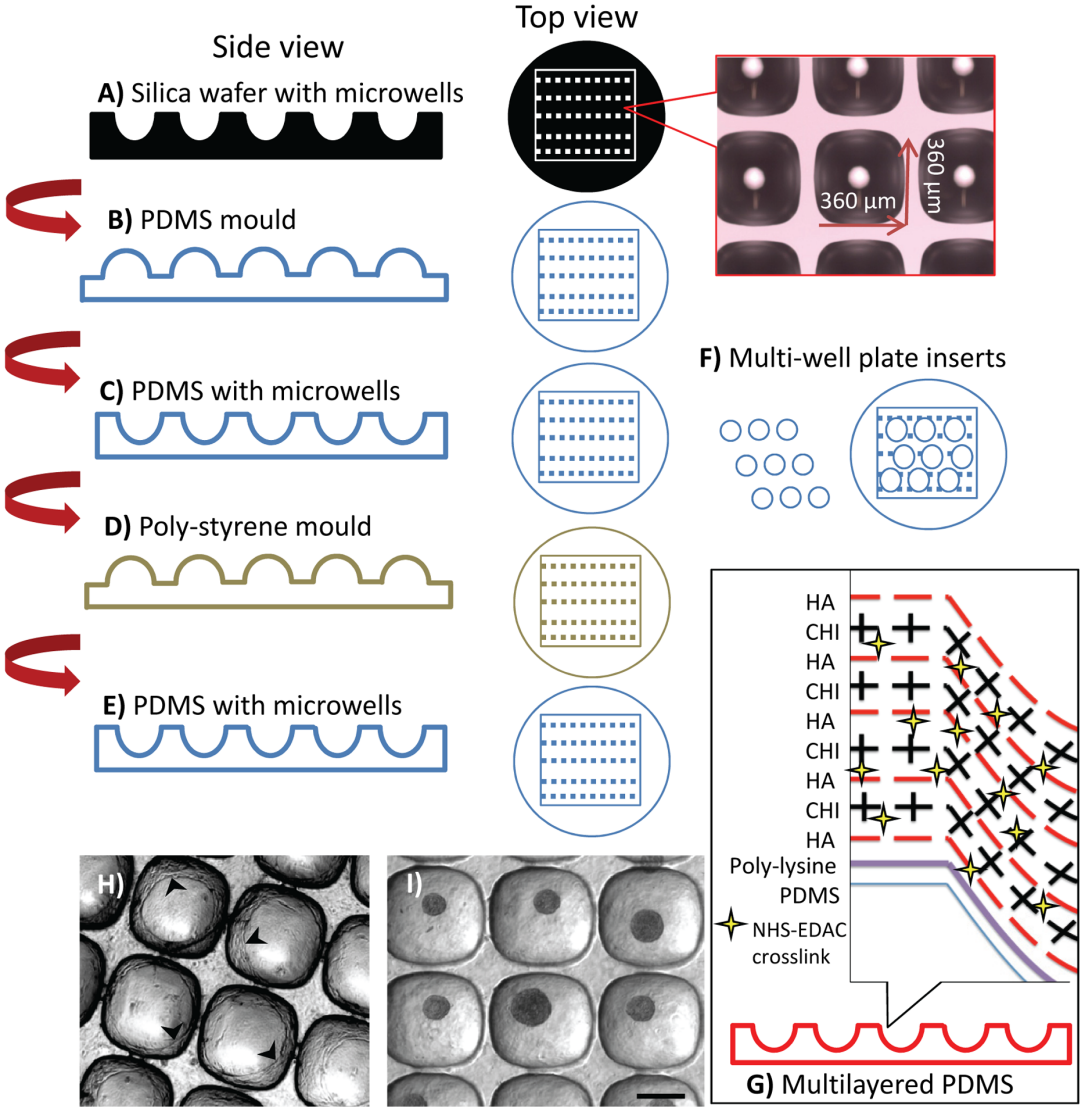
Fabrication of the microwell surface from PDMS replica moulding and surface modification. A silica wafer having an array pattern of microwells was formed via deep reactive ion etching. The dimensions of the microwells on silica wafer were 360×360×180 (depth) µm (A). This surface was used to cast PDMS, generating a negative surface. PDMS mould having an inverted microwell pattern (B). This surface was then coated in 5% pluronic acid solution, which functioned as a release agent. The coated surface was used to cast a 2 mm thick PDMS sheet having a microarray pattern identical to the original silica wafer (C). Because PDMS-PDMS casting was not reproducible, the PDMS sheet with the microwells was cast with a polystyrene sheet to obtain a plastic mould (D). Using polystyrene mould PDMS sheets with microwells were produced (E). A punch was used to create 2 cm^2^ discs which fit snuggly into the bottom of a 24-well plate (F). Individual microwell inserts were subsequently surface modified using a CHI/HA electrostatic multilayer; see text for details (G). The chondrocytes spreading on non-modified PDMS microwell surface (cell layers marked with arrowheads) (H). Robust micropellet formation on CHI/HA multilayered PDMS surface **(**I). Scale bar: 200 µm.

Soft lithography was used to prepare custom microwell surfaces [28]. A silica wafer having an array of microwells with the dimensions of 360 µm×360 µm×180 µm (Fig. 1A) was prepared via deep reactive ion etching [29] by the Australian National Fabrication Facility-Queensland (ANFF-Q). PDMS (Slygard®, silicone elastomer kit) was used to generate a negative imprint of the microwell surface on the silica wafer (Fig. 1A, B), as per the manufacturer’s protocol. This PDMS negative was then used as a mold to generate a replica of the original microwell surface. First, the PDMS negative was coated with a 5% solution of Pluronic-F127 to act as a release agent. The Pluronic-F127 coated surface was permitted to air dry over night. It was then used to cast the replica surface in a 2 mm thick layer of PDMS (Fig. 1C). This layer was cured at 60°C for one hour and then pealed from the negative. As this process was not sufficiently reproducible to enable reliable mass production of such surfaces, a hot embosser was used to cast this replica PDMS shape into polystyrene. This polystyrene surface then functioned as a mold that could be used repeatedly to cast microwell sheets (Fig. 1D, E). The polystyrene hot embossing was achieved by taking a sheet of polystyrene cut from a culture flask and pressing the PDMS replica surface into it (at 160°C for 15 minutes with minimal pressure, POWER TEAM hydraulic heat press), PDMS sheets with microwells were mass-produced (Fig. 1E), and 2 cm^2^ disks were punched from the PDMS sheets (Fig. 1F), which were inserted into 24-well plates (Nunc™).

### Surface Modification of Microwells

We previously showed that cells cultured on unmodified PDMS microwell surfaces have a propensity to spread rather than form pellets [30], and this microwell platform exhibited similar problems when culturing chondrocytes in serum-free chondrogenic redifferentiation medium (Fig. 1H). To minimize cell attachment to the microwell surface (Fig. 1I), the PDMS insert surface was chemically modified using a variation of our electrostatic multilayer (ML) technique [31] (Fig. 1G). Prior to ML deposition, a net negative charge was imparted on the PDMS microwell surface utilizing a hand-held high frequency plasma generator (Model BD-20, ETP) [32]. Immediately following plasma modification, the inserts were submerged in an electropositive poly-L-lysine solution (50 µg/mL in MES buffer, pH 5.5) and centrifuged at 4000×g for 5 minutes to ensure that the fluid entered the microwells. The poly-L-lysine was adsorbed onto the PDMS surface for 30 minutes at room temperature (RT). The wells were then rinsed twice with MES buffer, and ML deposition was initiated by adsorbing electronegative hyaluronic acid (HA) (50 µg/mL in MES buffer, pH 5.5) plus 1∶100 dilution of fresh cross-linker stock. N-Hydroxysuccinimide (NHS) and *N*-Ethyl-*N*′-(3-dimethylaminopropyl) carbodiimide hydrochloride (EDAC) cross-linker stock solution contained 50 mg/mL EDAC plus 70 mg/mL NHS in DMSO. We found that the DMSO stock solution could be effectively frozen and stored at −20°C in aliquots as long as the aliquots were used immediately upon thawing. The HA layers were adsorbed and cross-linked to the poly-L-Lysine for 20 minutes, and then the surfaces were washed twice with MES. Next, a layer of electropositive chitosan (CHI, 50 µg/mL CHI in MES buffer) was adsorbed to the HA layer for 20 minutes at RT. This process was repeated until 4 bilayers of HA-CHI were deposited, with the top layer being HA. The inserts were then sterilized overnight in 70% ethanol, washed three times with PBS, placed into sterile 24-well cell culture plates and kept hydrated in PBS at 4°C overnight.

The stability of the multilayer was tested by incubating multilayered and non-multilayered flat PDMS disks under different conditions and assessing cell attachment. Briefly, disks were incubated in 100% ethanol, 70% ethanol, acetone, liquid nitrogen, air, distilled water, PBS, boiling water for 24 hours, then ventilated for 15 minutes. The cells were seeded at a density of 3000 per cm^2^, incubated overnight in chondrogenic redifferentiation media. The next day, cell attachment was assessed (Fig. S1). For further information regarding characteristics of HA-CHI multilayer please see references [33], [34].

### Human Articular Chondrocyte Isolation and Expansion

Articular chondrocytes were isolated from intact articular cartilage tissue remaining on the knee joints donated following total joint replacement surgery. Ethical approval for this tissue recovery was granted through the Queensland University of Technology Ethics Committee and the Prince Charles Hospital in accordance with the Australian National Health and Medical Research Council’s Statement on Ethical Conduct in Research Involving Humans. Articular cartilage was minced into 3–4 mm pieces using a sterile scalpel. Tissue pieces were washed 3 times in phosphate-buffered saline (PBS; Gibco®). Pieces were suspended in 200 U/mL of Collagenase (Gibco®) diluted in low glucose Dulbecco’s modified Eagle’s medium (DMEM-LG; Gibco®), and then incubated overnight at 37°C. The digest was filtered through a 40 µm cell strainer (BD Falcon™) to separate tissue fragments. The filtered suspension was washed 3 times, each in 10 mL of DMEM-LG.

Chondrocytes were expanded in monolayer using T175 cm^2^ culture flasks (Nunc™) in 35 mL/flask volume of medium composed of DMEM-LG (Gibco®) supplemented with 10% fetal bovine serum (FBS; Gibco®), 100 U/mL penicillin and 100 µg/mL streptomycin (1% PS, Gibco®), 1% Glutamax (Gibco®), 40 µM ascorbic acid 2-phosphate, 40 µg/mL L-proline, in a humidified incubator having a 2% O_2_ and 5% CO_2_ atmosphere at 37°C. For the first two passages, 50 µg/mL gentamicin (Amersham Biociences©) and 2% PS were added to the medium. When monolayer cultures approached 80% confluence, the cells were harvested via 5-minute incubation with 3 mL 0.25% trypsin (Trypsin-EDTA; Gibco®) at 37°C. To inactivate the trypsin, 9 mL of expansion media containing 10% FBS and 1% PS was added. The cell suspension was centrifuged at 500×g for 5 minutes, the supernatant was discarded, and the cells were diluted into 3 times the previous growth medium volume and seeded into 3 T175 cm^2^ flasks, giving a split ratio of 1∶3. The experiment was repeated with three different donor chondrocytes, and passage 3 cells were used.

### Chondrogenic Redifferentiation Medium

Chondrogenic redifferentiation medium was composed of high-glucose DMEM (DMEM-HG; Gibco®), 10 ng/mL recombinant human Transforming Growth Factor- β1 (TGF-β1, Gibco®), 10^−7^ M dexamethasone, 200 µM ascorbic acid 2-phosphate, 100 µg/mL sodium pyruvate, 40 µg/mL L-proline, 1% ITS-X (Gibco®) and 1% PS.

### Formation of Macropellets

Macropellets were formed using a conventional pellet culture method [27]. In brief, 2×10^5^ cells were suspended in 1 mL of chondrogenic induction medium, then centrifuged in a 15 mL tube (LabServ®) at 500×g for 5 minutes, and then placed into a 2% or 20% O_2_–5% CO_2_ cell culture incubator at 37°C with the tube lid loosened to facilitate gas exchange.

### Formation of Micropellets

Micropellets were formed as described previously [27], [30], [35]. This design was modeled after work described by Ungrin *et al.* [36]. In brief, microscopic pellets of approximately 166 cells were formed using the patterned surface having 600 microwells/cm^2^ (described in Fig. 1). Approximately 1200 micropellets were formed from 2×10^5^ cells by suspending these cells in 1 mL of chondrogenic induction medium over 2 cm^2^ microwell inserts in the bottom of 24-well plates. Plates were centrifuged for 5 minutes at 500×g to facilitate pellet formation. Following centrifugation, an even distribution of cells within the microwells was confirmed via microscopy, and the plates were carefully transferred to a cell culture incubator set at 5% CO_2_ and either 2% or 20% O_2_ at 37°C.

### Assembly of Micropellets

The micropellets were transferred into a 15 mL tube then centrifuged at 500×g for 5 minutes to facilitate assembly after the culture of discrete micropellets, in microwells, for 4, 7, 11 or 14 days. The total culture time, including both micropellet culture and assembled culture, was 21 days. This part of the study was performed in only 2% O_2_ and 5% CO_2_ atmosphere at 37°C.

### Sulfated Glycosaminoglycan (sGAG) Quantification

Chondrogenic medium was exchanged twice weekly, and the medium from the macropellet and micropellet cultures was collected and stored at −80°C. When the culture was terminated, the recovered macropellets and micropellets were digested by adding 25 µg papain per sample directly to each tube or microwell plate then the pellet/enzyme mixtures were incubated at 60°C overnight. DMB dye (1,9-Dimethyl-Methylene Blue zinc chloride double salt) was used to quantify the sGAG content in the collected media and digested tissues using an established protocol [37]. In brief, a 30 µL volume of each sample of was dispensed into a single well of a 96-well clear plate (Nunc™), followed by the addition of 170 µL of DMB dye. The amount of sGAG was quantified by measuring the blue to purple color shift at 530 nm and 590 nm, respectively, in a plate reader (Benchmark Plus plate reader, Bio-Rad). Shark cartilage extract was used to generate a standard curve.

### Metabolic Activity Assay

AlamarBlue® (Invitrogen™) was used to assess the metabolic activity of the pellets as per the manufacturer’s protocol. The 10X alamarBlue® solution was diluted in the culture media of the pellets and incubated for 3 hours. Then the medium was removed and analyzed in a plate reader (POLARstar OPTIMA, BMG Labtech) at an excitation and emission of 544 nm and 590 nm, respectively.

### DNA Quantification

A Quant-iT™ PicoGreen® dsDNA Reagent and Kit (Invitrogen™) was used to determine DNA content in the cultures, as per the manufacturer’s protocol. In brief, 50 µL of papain digest was mixed with 50 µL of PicoGreen dye in a fluorescence plate (Nunc™) and analyzed in a plate reader (POLARstar OPTIMA, BMG Labtech) at an excitation and emission of 480 nm and 520 nm, respectively.

### Relative Gene Expression Analysis

TRIzol® (Invitrogen™) was used for RNA extraction, as per the manufacturer’s protocol. RNA concentration was determined using a Nanodrop ND-1000 spectrophotometer (Bio-Lab). cDNA was synthesized from RNA template using SuperScript III RT and oligo(dT)20 (Invitrogen™) as per the manufacturer’s protocol, and stored at −80°C until analysis. Real-time polymerase chain reaction (qPCR) was performed using Platinum® SYBR® Green qPCR SuperMix-UDG (Invitrogen™) using the primer sequences shown in Table S1 (Geneworks). The master mix was dispensed into the 384-well reaction plate and combined with cDNA samples using an epMotion 5057 (Eppendorf) liquid handling robot. The plates were processed in a 7900HT Fast Real-Time PCR System (Applied Biosystems). PCR cycling parameters were 50°C for 2 minutes, 95°C for 3 minutes, 95°C for 15 seconds and 60°C for 30 seconds, repeated for a total of 40 cycles. The results were analyzed using the ΔΔCt method normalized to the geometric mean of two housekeeping genes (cyclophilin A and glyceraldehyde 3-phosphate dehydrogenase (GAPDH)) [38].

### Histological Analysis

Harvested tissues were embedded in optimum cutting temperature compound (OCT, Tissue-Tek®), and stored at −80°C. 10 µm-thick sections of samples were generated using a microtome-cryostat (Leica®), and then adsorbed onto poly-lysine glass slides (Thermo Fisher Scientific) and stored at −20°C until further analysis.

For Alcian blue staining, the slides were fixed with 4% paraformaldehyde for 20 minutes at RT then rinsed with PBS 3 times. Following rinsing, slides were dried and the sections submerged in fresh filtered 1% Alcian blue solubilized in 3% acetic acid (pH 2.5) for 10 minutes. The slides were then rinsed thoroughly with PBS and observed under Laborlux S microscope (Leitz®) using bright field illumination. For immunofluorescence (IF), the slides were fixed with 4% paraformaldehyde for 20 minutes at RT, and then rinsed with PBS 3 times. The slides were dried and borders drawn around sections using a PAP pen. The sections were blocked (3% goat serum, 0.3% Triton X-100 in 1% BSA/PBS) for 20 minutes at RT. The blocked sections were incubated with collagen I, II and X primary antibodies (raised in mouse, rabbit and rabbit respectively, Abcam®) at 4°C overnight in a humidity chamber. The slides were washed with 0.3% Triton X-100 in PBS for 3 minutes, and then rinsed with PBS. The sections were incubated with corresponding secondary antibodies (FITC conjugated anti mouse IgG2b and Cy-3 conjugated anti rabbit IgG, Abcam®) for 30 minutes at RT. Slides were washed twice with 0.3% Triton X-100 in 1% BSA/PBS and once with PBS. Coverslips were mounted onto slides using the ProLong Gold Antifade Reagent (Invitrogen™), and assessed under an Eclipse TE2000-U (Nikon) fluorescence microscope using NIS Elements (F 3.2) software.

### Statistical Analysis

All experiments contained *n  = 4* biological replicates. Studies were repeated using chondrocytes derived from three donors. Data were represented as mean ± standard deviation. Data were analyzed using SPSS (statistical software package: SPSS® Inc.) and one-way analysis of variance (ANOVA) with Tukey post-hoc tests to identify statistical significance, (*) represents *p<0.05* and (***) represents *p<0.001*.

## Results

### Morphology and Size of the Pellets

**Figure 2:**
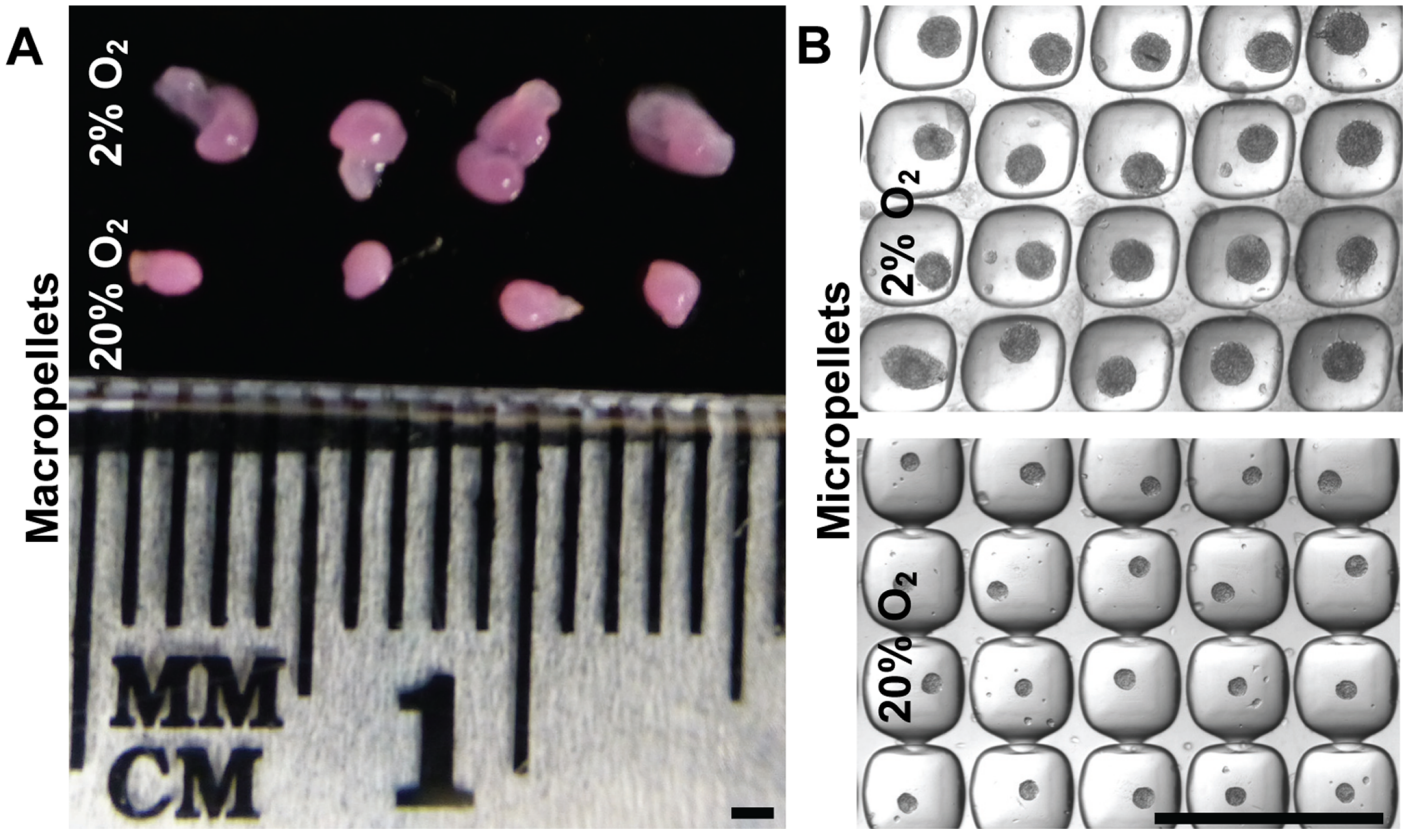
Morphology and size of the pellets. At the end of the 14-day culture, both hypoxic macropellets (A) and micropellets (B) were bigger than the normoxic pellets. The estimated mean diameter for hypoxic micropellets was 193±20 µm (*n  = 20*) whilst the estimated mean diameter of the normoxic micropellets was 87±10 µm (*n  = 20*). Scale bars: 1 mm.

Chondrocytes aggregated into micropellets within 24 hours (data not shown). At day 14 of culture, both hypoxic macropellet and micropellet diameters were greater than normoxic pellet diameters (Fig. 2A, B). The estimated average diameter of the hypoxic micropellets was 193±20 µm (*n  = 20*), whilst the normoxic micropellets had an estimated average diameter of 87±10 µm (*n  = 20*).

### Metabolic Activity, DNA and sGAG Production

**Figure 3:**
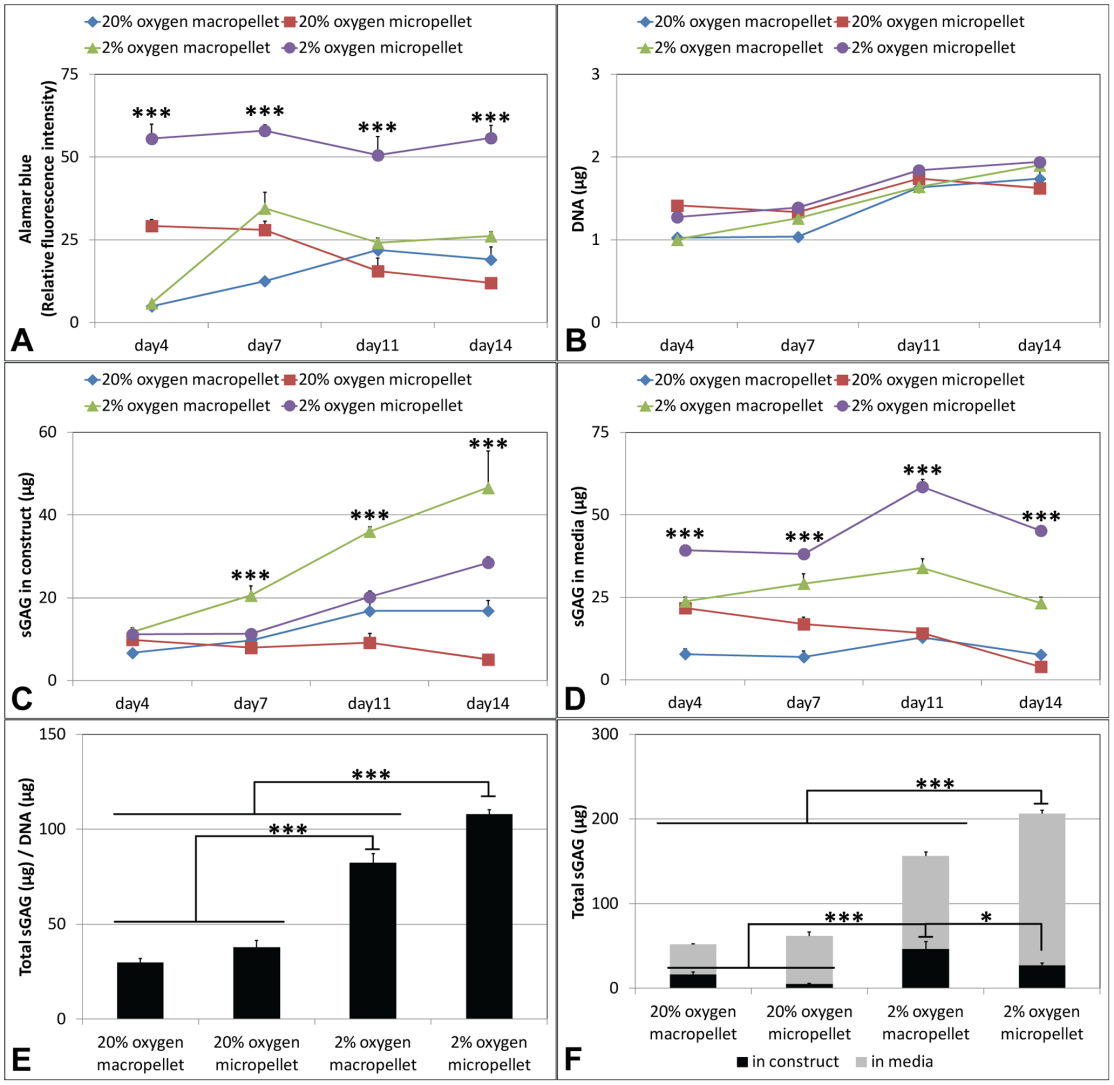
Metabolic activity, growth and sGAG production in pellets. AlamarBlue® graph for metabolic activity (A), DNA quantification (B), sGAG in construct (C) and sGAG in media (D) measurements on days 4, 7, 11, 14. The sGAG/DNA ratio (calculated by dividing the total amount of sGAG produced during the culture to the amount of DNA measured on day 14) (E) and the total sGAG graph demonstrating the total sGAG in media and in construct separately (F).

Hypoxic micropellets were significantly more metabolically active than the other pellets over the culture period, as assessed by alamarBlue® (Fig. 3A). DNA quantification demonstrated that the proliferation of the cells did not differ significantly in different conditions (Fig. 3B). To assess the recovery of the chondrogenic phenotype, the amount of sGAG secreted into the medium over the culture duration and in the final tissues was quantified. The amount of the sGAG retained inside the pellet was significantly higher for the hypoxic macropellets at day 7, 11 and 14 (Fig. 3C). However, the amount of sGAG released into the media was highest for the hypoxic micropellets at all time points (Fig. 3D). The sGAG/DNA ratio was calculated by dividing the total amount of sGAG produced during the culture to the amount of DNA measured at the end of the culture. Hypoxic micropellets had the greatest ratio when compared to other pellets; hypoxic macropellets also had a significantly higher ratio than normoxic pellets (Fig. 3E). The amount of total sGAG measured in the media was higher than the amount measured in the pellets for all conditions (Fig. 3F). The retained sGAG was the highest for the hypoxic macropellets, whilst the overall produced sGAG was greater for the hypoxic micropellets (Fig. 3F).

### Chondrogenic and Hypertrophic Gene Expression

**Figure 4:**
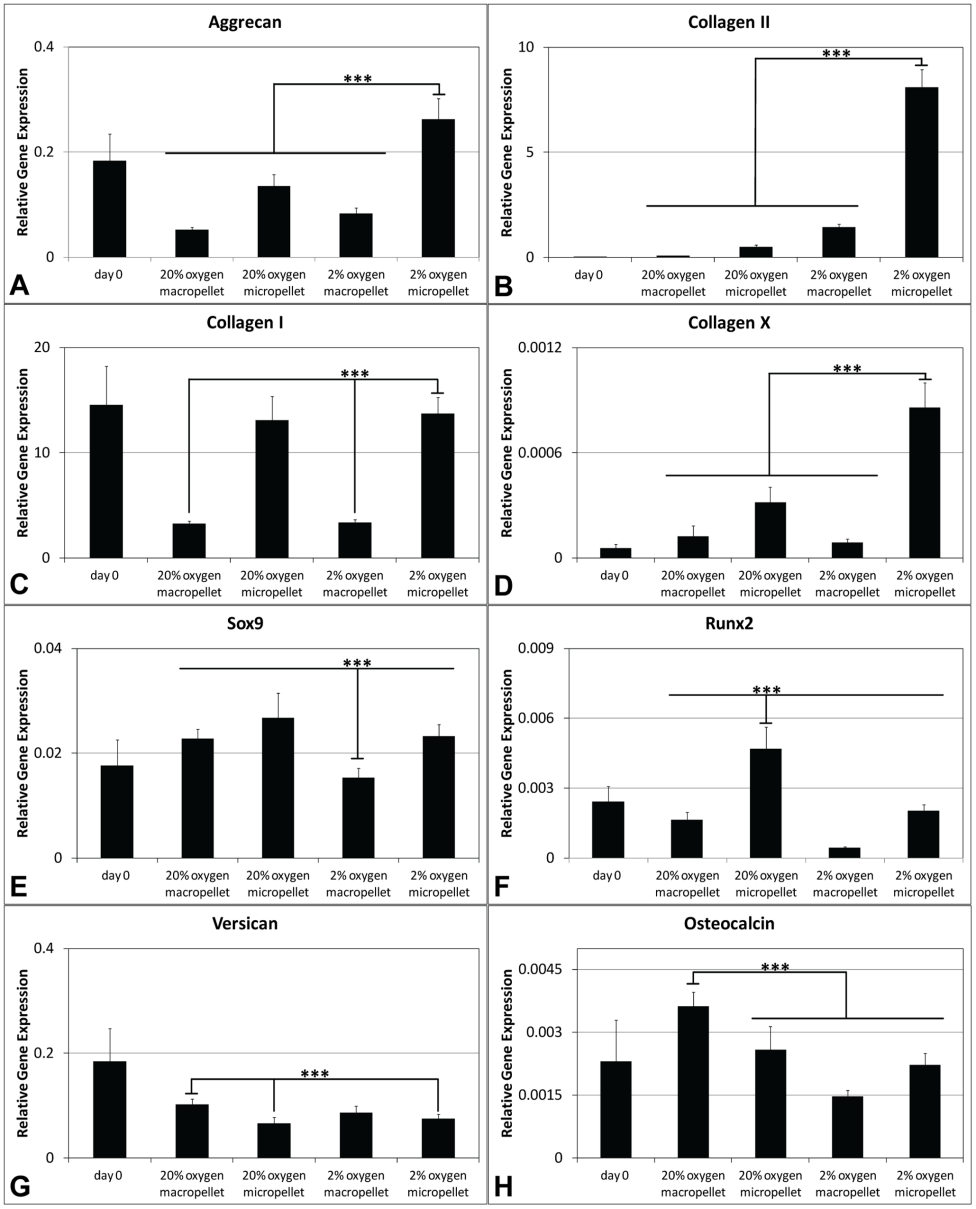
Gene expression in pellets. Aggrecan (A), collagen II (B), collagen I (C), collagen X (D), Sox9 (E), Runx2 (F), versican (G), and osteocalcin (H) expressions relative to the geometric mean of housekeeping genes cyclophilin A and GAPDH.

Chondrocyte-associated expression of Sox9, aggrecan and collagen II; and hypertrophy associated expression of Runx2, collagen I, collagen X, osteocalcin and versican [27] were assessed. Key matrix genes like aggrecan and collagen II had the highest expression in the hypoxic micropellets (Fig. 4A, B). Collagen I was significantly downregulated in macropellets, but remained unchanged in micropellet cultures relative to day 0 controls (Fig. 4C). Runx2 (Fig. 4F) expression was greater in normoxic micropellets, as was collagen X expression in hypoxic micropellets (Fig. 4D). Sox9 expression was lower in hypoxic macropellets (Fig. 4E). Macropellets maintained in a normoxic atmosphere had the highest expression of versican (Fig. 4G) and osteocalcin (Fig. 4F).

### Chondrogenic and Hypertrophic Matrix Deposition and Distribution

**Figure 5:**
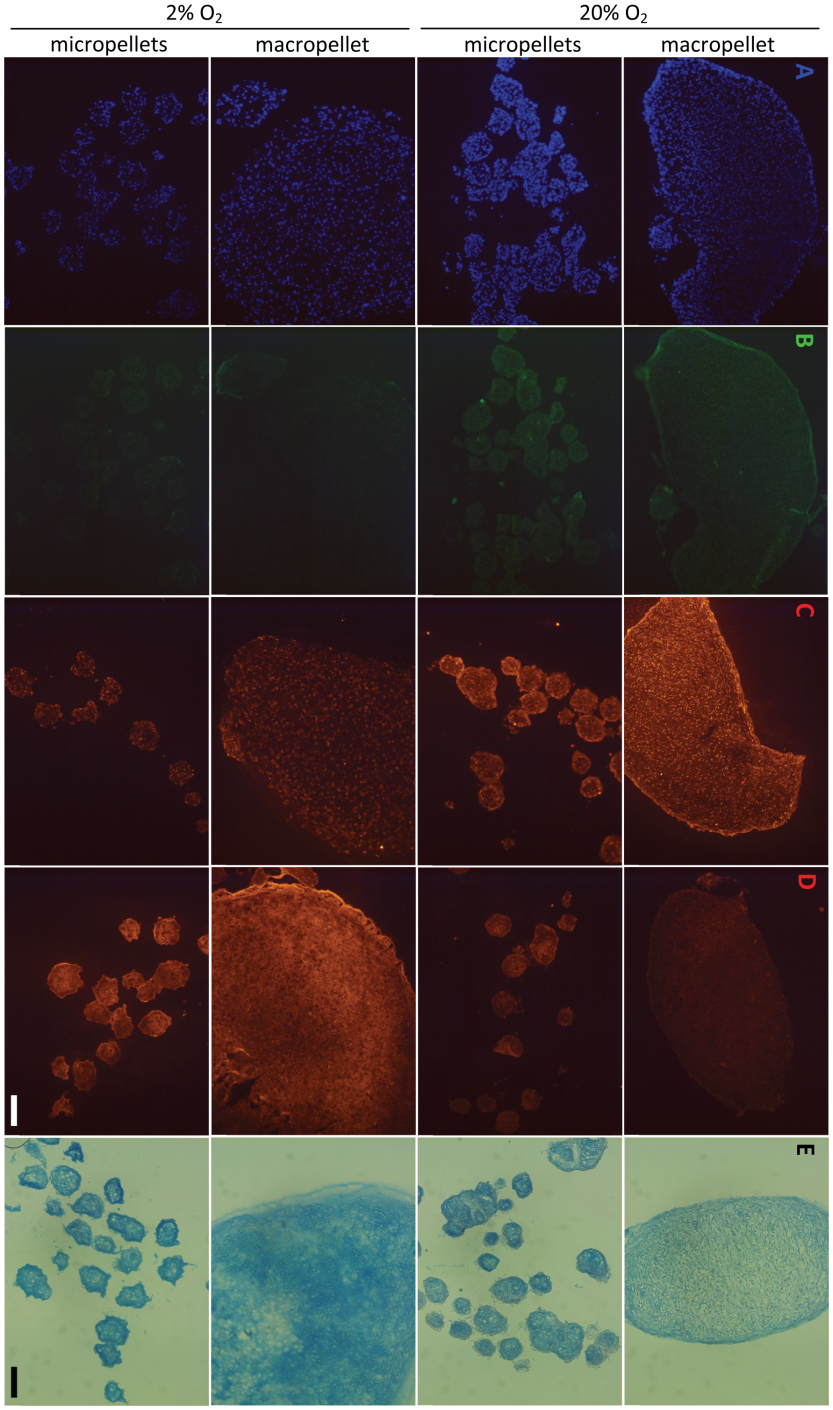
Cell and matrix localization throughout pellets following 14 days of culture. DAPI staining of nuclei in pellets (A).Immunofluorescence images for collagen I (B), collagen X (C), and collagen II (D), Alcian blue staining for sGAG (E). Scale bars: 100 µm.

To visualize the distribution of the ECM molecules within the macro and micropellets, Alcian blue staining and IF analysis for collagen I, II and X were performed. DAPI staining was used to visualize the nuclei (Fig. 5A). Collagen I accumulation was minimal in all conditions, but appeared even lower in hypoxic cultures (Fig. 5B). Collagen X was more intense in normoxic cultures relative to cultures maintained in hypoxic atmospheres (Fig. 5C). By contrast, collagen II staining was stronger in hypoxic cultures. Collagen II matrix distribution in hypoxic macropellets appeared non-uniform, whilst individual micropellets were stained more homogeneously (Fig. 5D). Alcian blue staining revealed that sGAG was lower in normoxic macropellets and staining was more homogeneous in micropellets relative to macropellets (Fig. 5E).

### Micropellet Assembly into Macrotissues

**Figure 6:**
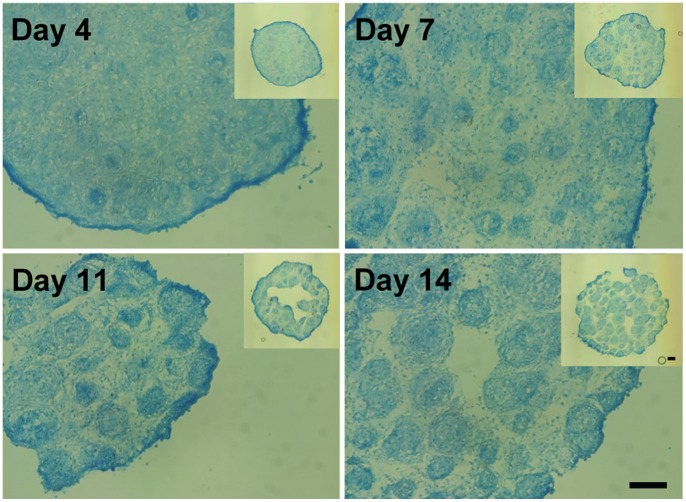
Hypoxic micropellets assembled into macrotissues. Alcian blue staining for hypoxic micropellets assembled at different time points (indicated days). The total culture duration was 21 days. Scale bars: 100 µm.

To assess the interaction between individual micropellets, they were collected from the microwell surface and centrifuged into a single aggregate at different time points during the chondrogenic redifferentiation process. Micropellets collected from day 4 cultures that were assembled into larger tissue constructs integrated in a uniform manner, such that discrete microtissues were virtually indistinguishable via Alcian blue staining. By contrast, it was still possible to identify individual micropellets collected from day 14 cultures that had been assembled into larger tissue constructs, indicating that full integration had not yet occurred in these constructs (Fig. 6).

## Discussion

A number of strategies are available to facilitate cell aggregate manufacture (reviewed in [39]), including hanging drop and various rotary bioreactors. Our group favors microwells, as they offer an unparalleled capacity to facilitate robust and precise high-throughput cell aggregate manufacture. Previous studies utilized a commercial microwell product (Aggrewell™, STEMCELL Technologies) to efficiently manufacture micropellets [27], [30], [35]. However, if the unmodified PDMS microwell surface is utilized directly in micropellet manufacture, the chondrocytes will adhere to the surface rather than form micropellets (Fig. 1H). In our studies, modifying the surface to prevent cell attachment further enhanced the performance of Aggrewell™. Minimizing cell attachment favoured aggregate formation and facilitated aggregate harvest. As a cost effective strategy, also to enable full control and to ease the optimization of the surface properties, we designed and fabricated our own microwell surface. Figure 1 outlines the fabrication process that was used to generate PDMS microwell inserts that fit into 24-well plates. Following surface modification with the HA/CHI ML (Fig. 1G), the microwell surface enabled robust chondrocyte micropellet formation and harvest (Fig. 1I). Additionally, the stability of the multilayer was tested, and the surface modification remained functional even after 24 hour incubation in 100% ethanol, 70% ethanol, liquid nitrogen, air, distilled water, PBS or boiling water (Fig. S1).

Other available microwell platforms are also suitable for chondrocyte micropellet manufacture. For example, the development of a microwell platform in which the microwells were cast in an agarose gel rather than PDMS was recently described [40]. In this clever strategy, the agarose surface promoted cell aggregate formation by resisting protein adsorption and subsequent cell attachment to the agarose surface. However, a more mechanically robust PDMS microwell platform is compatible with centrifugation, which enables more rapid and efficient micropellet manufacture than the gravity settling method required with an agarose platform. Additionally, there is comparatively less risk of damaging the PDMS microwell structure during medium exchange or culture manipulation processes.

**Figure 7:**
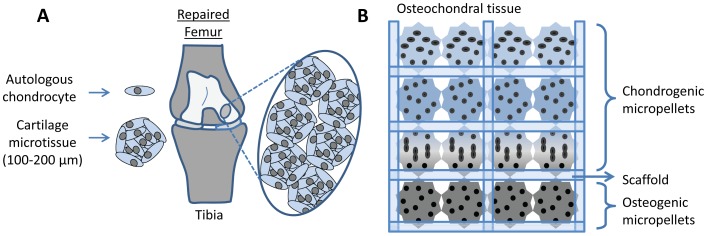
Potential applications of the chondrocyte micropellets. The direct use of chondrocyte micropellets in articular cartilage defect repair (A). The use of cartilage micropellets in the manufacture of osteochondral tissues *in vitro* (B).

Enhanced sGAG synthesis is critical for effective cartilage tissue regeneration, as the sGAG content endows cartilage with its compressive strength [27]. The sGAG outputs in both micropellet and macropellet cultures were significantly enhanced when these cultures were maintained in hypoxic atmospheres (Fig. 3E). These results are consistent with previous studies demonstrating that hypoxia enhances chondrocyte redifferentiation by stabilizing the hypoxia inducible factor 1 alpha (HIF1α), which is translocated into nucleus and activates chondrogenic gene expression [41], [42]. However, this is the first study comparing the sGAG production between micropellets and macropellets, under both hypoxic and normoxic environments. Consistent greater sGAG production is observed in hypoxic micropellets, whilst greater sGAG retention occurs in the hypoxic macropellets (Fig. 3F). The loss of the sGAG to the medium is a commonly reported challenge in cartilage tissue engineering, and solutions have been suggested in previous papers [43], [44]. The high surface-area-to-volume ratio in the micropellets contributes to the significant loss of sGAG to the medium. However the greater sGAG production and superior gene expression indicate that the chondrocyte redifferentiation was enhanced in hypoxic micropellets. If it is possible to improve the quality of the redifferentiation process, then this short term *in vitro* loss of sGAG should be insignificant relative to the overall clinical benefits. The retention of sGAG would likely be enhanced during a cartilage repair procedure where a large number of micropellets would be implanted into a sealed cartilage defect (Fig. 7A).

Consistent with the sGAG results, chondrogenic gene expression also indicated that the redifferentiation was enhanced in hypoxic micropellets. No significant difference was observed in hypoxic micropellet expression profile for collagen I, Sox9, Runx2, versican and osteocalcin when compared to day 0 measurements (Fig. 4C, E, F, G, H). The expression of some osteogenic genes was elevated in the day 0 cultures, and this reflects the fact that the chondrocytes used in this study were all derived from tissue discards harvested from elderly patients suffering from severe osteoarthritis. Whilst collagen X expression was significantly upregulated in hypoxic micropellets, the overall magnitude of the expression was very low (0.0012 times) relative to the expression of housekeeping genes (Fig. 4D). By comparison, collagen II gene expression was ∼10-fold greater in the hypoxic micropellet cultures than the housekeeping gene expression (Fig. 4B). Despite having some hypertrophic properties, the hypoxic micropellets exhibit superior redifferentiation when data are considered cumulatively. The hypoxic micropellets had a larger volume (Fig. 2B), greater metabolic activity, sGAG production (Fig. 3A, E) and higher collagen II expression (Fig. 4B). Importantly, the deposition of the collagen II and sGAG was more uniform in the micropellets relative to macropellets (Fig. 5D, E). More uniform cell behavior is consistent with a smaller diameter pellet with reduced diffusion gradients. These data mirror our previously reported results indicating that the micropellet strategy enhanced uniformity in MSC chondrogenic cultures [27].

Co.don® AG (Teltow, Germany) is currently evaluating the potential of macropellets for cartilage defect repair in on-going trials [25], [26]. We suggest that there may be legitimate benefits in using micropellets rather than macropellets, as this should provide for a more uniform and potent clinical product. Additionally, because of their smaller geometry, micropellets may be able to accommodate more complex defect geometries and ultimately produce a smoother articular surface. This is a rational expectation, as smaller diameter spheres will always more uniformly fill a void than larger diameter spheres. A prerequisite to such applications is that micropellets must demonstrate the capacity to amalgamate into a contiguous repair tissue. Here we tested the amalgamation efficiency of cartilage micropellets that had been cultured for 4, 7, 11 or 14 days. The amalgamation efficiency depended on the time that the micropellets had been cultured before assembly (Fig. 6), and the most primitive day 4 micropellets proved the most efficient. This outcome is also rational, as temporal matrix deposition in the micropellets would be expected to stabilize with time. These results reflect only short-term observation, and it may be possible to observe excellent integration of even day 14 micropellets over an extended time period. Given that micropellets contain significant matrix content, we reason that they may be superior to single cell suspensions lacking any legitimate matrix component, as in procedures like Autologous Chondrocyte Implantation (ACI). Unlike macropellets, micropellets could easily be injected under ACI-type membranes, thus facilitating delivery.

Using our microwell system, it is possible to generate 36,000 cartilage micropellets in a single 6-well plate. This manufacturing efficiency enables the evaluation of micropellets either in the direct repair of cartilage defects (Fig. 7A), or as a building block in the *in vitro* assembly of complex zonal osteochondral tissues (Fig. 7B). Typically, osteochondral tissues have been made from single cell suspensions seeded into gels or onto solid scaffolds [45], [46], [47], [48], although a recent study used spheroids of rabbit MSCs differentiated into osteoblasts and chondrocytes, which were subsequently assembled into a zonal tissue [49]. In this study, the spheroids were manufactured by manually dispensing cells in collagen droplets. Our more efficient manufacturing process should enable more sophisticated zonal tissues such as those shown schematically in Figure 7B. Data from our group indicated that a similar micropellet manufacturing strategy enabled enhanced MSC osteogenesis and the generation of bone spheroids [35] ideal for the assembly of an osteochondral tissue. Ultimately, the small dimensions of micropellets make them ideal for identifying the culture conditions necessary to recapitulate the various zonal tissues found in cartilage [50], and therefore, micropellets will likely become the preferred building blocks for reconstructing such tissues.

### Conclusion

Herein we describe the fabrication of a custom microwell system and a surface modification that enables the efficient manufacture of thousands of cartilage micropellets. Hypoxic micropellet culture was shown to be a superior chondrocyte redifferentiation platform, relative to traditional macropellet cultures. We rationalized that the micropellets might offer a unique strategy for enhanced cartilage defect repair, and to investigate this potential the efficiency of the micropellet amalgamation was examined. Micropellets that had been cultured for 4–7 days most efficiently amalgamated, and the composite tissue was nearly seamless at the end of the 21-day culture. Cumulatively, our results indicate that the redifferentiation of expanded human articular chondrocytes can be enhanced using micropellet culture, and that these micropellets can be assembled into larger more clinically relevant dimensions.

## Supporting Information

Figure S1 **Surface modification testing.** To assess the stability and functionality of the surface modification after incubation in ethanol and PBS, a testing platform was set up as follows: flat 24 well plate PDMS disks were produced and half of them were multilayered as explained in the Materials and Methods section. The disks were incubated under conditions stated (in 100% ethanol, in 70% ethanol, in acetone, in liquid nitrogen, in air, in distilled water, in PBS, in boiling water) for 24 hours and the functionality of the surface was assessed by imaging cell attachment. After 15 minutes of ventilation, cells were seeded at a density of 3000/cm^2^, incubated in chondrogenic redifferentiation media overnight. Surface modification was not affected by any of the conditions. However acetone sensibly decreased the transparency of the PDMS itself therefore the cell attachment could not be assessed. For all other conditions the cell spreading was observed for the surface with no multilayer whereas the cells were not spreading on the surfaces with multilayer.
(TIF)

Table S1 **Primers used for gene expression analysis.**
(DOCX)
