# Supplementary figures and images for: The Interplay between Chondrocyte Redifferentiation Pellet Size and Oxygen Concentration
Source: PLoS One. 2013 Mar 15;8(3):e58865. doi: 10.1371/journal.pone.0058865 (PMC3598946; doi:10.1371/journal.pone.0058865)

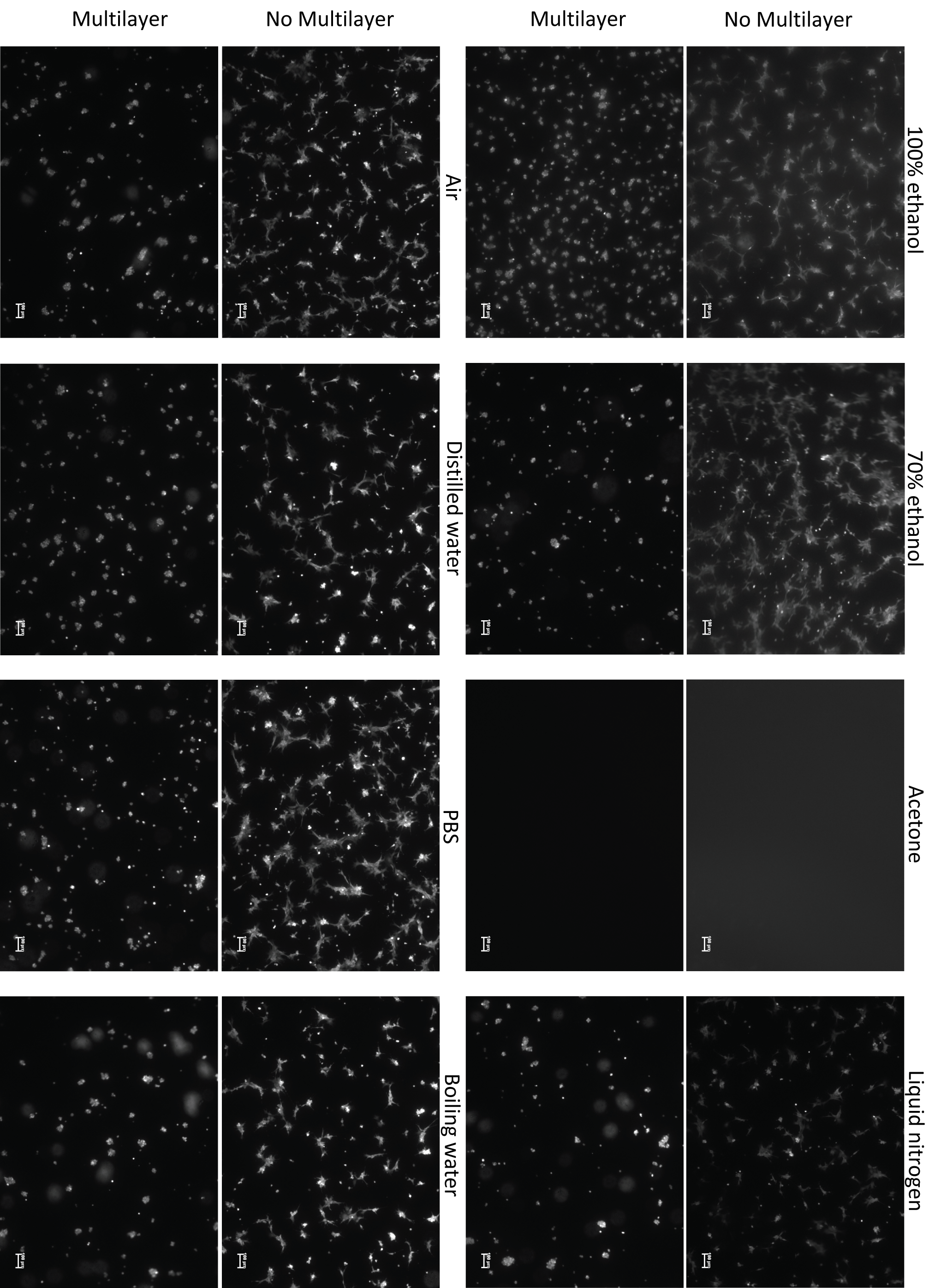

Supplement: Figure S1 — Surface modification testing. To assess the stability and functionality of the surface modification after incubation in ethanol and PBS, a testing platform was set up as follows: flat 24 well plate PDMS disks were produced and half of them were multilayered as explained in the Materials and Methods section. The disks were incubated under conditions stated (in 100% ethanol, in 70% ethanol, in acetone, in liquid nitrogen, in air, in distilled water, in PBS, in boiling water) for 24 hours and the functionality of the surface was assessed by imaging cell attachment. After 15 minutes of ventilation, cells were seeded at a density of 3000/cm2, incubated in chondrogenic redifferentiation media overnight. Surface modification was not affected by any of the conditions. However acetone sensibly decreased the transparency of the PDMS itself therefore the cell attachment could not be assessed. For all other conditions the cell spreading was observed for the surface with no multilayer whereas the cells were not spreading on the surfaces with multilayer. (TIF) [file pone.0058865.s001.tif]
