# Supplementary material for: The Interplay between Chondrocyte Redifferentiation Pellet Size and Oxygen Concentration
Source: PLoS One. 2013 Mar 15;8(3):e58865. doi: 10.1371/journal.pone.0058865 (PMC3598946; doi:10.1371/journal.pone.0058865)
Supplement: Table S1 — Primers used for gene expression analysis. (DOCX) [file pone.0058865.s002.docx]

| **Gene (Amplicon size in basepair)** | **Primers** |
| --- | --- |
|  |  |
| Cyclophilin A (164) | Forward CTCGAATAAGTTTGACTTGTGTTT |
|  | Reverse CTAGGCATGGGAGGGAACA |
| GAPDH (119) | Forward ATGGGGAAGGTGAAGGTCG |
|  | Reverse TAAAAGCAGCCCTGGTGACC |
| SOX9 (77) | Forward TTCCGCGACGTGGACAT |
|  | Reverse TCAAACTCGTTGACATCGAAGGT |
| Aggrecan (85) | Forward TCGAGGACAGCGAGGCC |
|  | Reverse TCGAGGGTGTAGCGTGTAGAGA |
| Collagen II, COL2A1 (79) | Forward GGCAATAGCAGGTTCACGTACA |
|  | Reverse CGATAACAGTCTTGCCCCACTT |
| Collagen I, COL1A1 (83) | Forward CAGCCGCTTCACCTACAGC |
|  | Reverse TTTTGTATTCAATCACTGTCTTGCC |
| Versican (98) | Forward TGGAATGATGTTCCCTGCAA |
|  | Reverse AAGGTCTTGGCATTTTCTACAACAG |
| Collagen X, COL10A1 (70) | Forward CAAGGCACCATCTCCAGGAA |
|  | Reverse AAAGGGTATTTGTGGCAGCATATT |
| Runx2 (113) | Forward GGAGTGGACGAGGCAAGAGTTT |
|  | Reverse AGCTTCTGTCTGTGCCTTCTGG |
| Osteocalcin (70) | Forward GAAGCCCAGCGGTGCA |
|  | Reverse CACTACCTCGCTGCCCTCC |
